# Dietary patterns are associated with type 2 diabetes mellitus among middle-aged adults in Zhejiang Province, China

**DOI:** 10.1186/s12937-017-0303-0

**Published:** 2017-12-13

**Authors:** Long Shu, Xiao-Ming Shen, Chun Li, Xiao-Yan Zhang, Pei-Fen Zheng

**Affiliations:** 1 Department of Nutrition, Zhejiang Hospital, Lingyin Road Number 12, Xihu District, Hangzhou, 310013 Zhejiang People’s Republic of China; 2 Department of Endocrinology, Pinghu First People’s Hospital, Pinghu, 314200 Zhejiang People’s Republic of China; 3 Department of Digestion, Zhejiang Hospital, Lingyin Road Number 12, Xihu District, Hangzhou, 310013 Zhejiang People’s Republic of China

**Keywords:** Dietary patterns, Factor analysis, Type 2 diabetes mellitus, Middle-aged Chinese, Nutritional epidemiology

## Abstract

**Background:**

Although some studies have shown the associations between dietary patterns and the risk T2DM in a general population, the associations in middle-aged Chinese have been rarely studied to date. In this study, we aimed to characterize dietary patterns in Chinese adults aged 45-59y (*n* = 1918) and to evaluate the relationship between dietary patterns and the risk of T2DM.

**Methods:**

The study population was a part of the population-based the Nutrition and Health Study conducted in the city of Hangzhou, Zhejiang Province, China. Dietary intake was assessed by using a validated food frequency questionnaire (FFQ). Multivariate logistic regression analyses were used to estimate the associations between dietary patterns and the risk of T2DM, adjusting for potential confounders.

**Results:**

Three major dietary patterns were identified using factor analysis, including the traditional southern Chinese, the Western, and the grains-vegetables patterns. After adjusting for the potential confounders, subjects in the highest quartile of the Western dietary pattern scores had greater odds ratio(OR) for T2DM(OR = 1.28; 95% confidence interval (CI): 1.103–1.697; *P* = 0.02) than did those in the lowest quartile. Compared with those in the lowest quartile, subjects in the highest quartile of the grains-vegetables dietary pattern scores had a lower OR for T2DM (OR = 0.72; 95% CI:0.596–0.952; *P* = 0.04). Moreover, no significant association was found between the traditional southern Chinese dietary pattern and risk of developing T2DM.

**Conclusions:**

Our findings indicated that the Western dietary pattern was associated with an elevated risk, whereas the grains-vegetables dietary pattern was associated with a reduced risk of T2DM. Further researches are needed to confirm these findings.

## Background

The prevalence of T2DM is rapidly increasing worldwide, accounting for 90% of diabetes [1]. In Asia, the prevalence of T2DM has increased from 1.2% in 1994 to 14.6% in 2003 [2]. In China, T2DM has been recognized as a major health problem and the prevalence has also increased considerably from 13.7% between 2000 and 2001 to 27.4% between 2009 and 2010 [3]. It is well-known that T2DM is recognized as a multifactorial chronic disease that can be related to unhealthy lifestyles, e.g. physical inactivity and smoking, genetic factors and dietary factors [4, 5].

Over the past several decades, epidemiological studies [5–7] have shown that diet plays an important role in the development of T2DM, and reported the association between the intake of individual foods or nutrients and the risk of T2DM [6, 7]. However, due to the complexity of diets and the potential interactions between food components [8], these analyses revealed limited impact of diets on T2DM. Consequently, dietary pattern analysis becomes a more recognizable approach because it reflects the complexity of dietary intake and can potentially facilitate nutritional recommendations [9].

Recently, there has been considerable attention in nutritional epidemiology towards the associations between dietary patterns and the risk of T2DM [10–12]. However, to date, data on the associations between dietary patterns and T2DM in the Chinese population are limited [13, 14], particularly in a middle-aged population. To the authors’ knowledge, no previous epidemiological studies have reported the associations between dietary patterns and risk of T2DM in a middle-aged population in China. Therefore, in the present study, we aimed to characterize dietary patterns in Chinese adults aged 45-59y and to evaluate the associations between dietary patterns and the risk of T2DM.

## Subjects and methods

### Study population

This cross-sectional study was conducted in Hangzhou city, the capital of Zhejiang Province, East China, from June 2015 to December 2016. The study sample was taken from ten areas(Xihu, Gongshu, Shangcheng, Xiacheng, Bingjiang, Jianggan, Xiaoshan, Yuhang, Fuyang and Linan) and three counties (Tonglu, Chunan, Jiande) by a stratified cluster random-sampling method. We chose two residential village or community from every county or area randomly, according to resident health records, with participants aged 45–59 years residing in the selected villages or communities. A total of 2437 eligible participants were invited to attend a health examination at the Medical Center for Physical Examination, Zhejiang Hospital, where participant were interviewed face-to-face by a trained interviewer using written questionnaires. After exclusion of 124 participants with family history of diabetes, cardiovascular disease or stroke, 2313 participants remained for this study. Moreover, we further excluded 236 participants who provided missing information on dietary intake, 20 participants who didn’t provide blood sample, 112 participants who provided incomplete anthropometric information and 27 participants who self-reported a history of taking medications(these medications may affect serum lipoprotein concentrations, blood pressure and carbohydrate metabolism). Finally, 1918 participants (996 males) remained for the present analysis of the associations between dietary patterns and the risk of T2DM (Appendix 1).

### Assessment of dietary intake

Dietary information were collected using a food frequency questionnaire (FFQ) previously validated [15]. The validity of the FFQ was assessed by a pilot survey within the same population by comparison with three 24-h dietary recalls before the study, and the correlation coefficients between the FFQ and averages from 24-h dietary recalls ranged between 0.43 and 0.67 for major food groups. In general, these data showed that the FFQ provided reasonably valid measurements of dietary intakes.The FFQ consisted of 138 food items with standard portion sizes commonly consumed by Chinese. Participants were asked to recall the frequency of each food item over the previous 12 months and the estimated portion size, using local weight units (1 Liang = 50 g) or natural units (cups). The frequency of each food item was classified as follows: never, less than 1 time/month, 1 to 3 times/month, 1 to 2 times/week, 3 to 4 times/week, 5 to 6 times/week, 1 time/day, 2 times/day, and 3 times/day. Finally, data was converted into grams(g)/day or ml/d in the following analysis.

### Identification of dietary patterns

First, we aggregated 138 food items from the the FFQ into 30 food groups (in Table 1) based on their similarity in nutrients and earlier studies [15] in China.We then conducted the factor analysis (principal component) on the 30 food groups to identify dietary patterns. The obtained factors were rotated using orthogonal rotation so that the factors(dietary patterns) were uncorrelated with each other and were easier to interpret.The eigenvalue and scree plot were applied to decide which factors remained [16]. After evaluating the eigenvalues, the scree plot test, and interpretability, factors with an eigenvalues ≥ 2.0 were retained. Individual food items with a factor loading ≥ |0.4| were considered to significantly contribute to the pattern in this study. The labeling of dietary patterns was based on the interpretation of foods with high factor loadings for each dietary pattern [17]. Factor scores were categorized into quartiles (Q1 represented a low intake of the food pattern; Q4 represented a high intake of the food pattern).

**Table 1:** Food grouping used in the dietary pattern analyses

Food groups	Food items
Refined grains	Rice, porridge, rice in soup, noodles,instant noodles,steamed bun, wonton, dumplings,white breads, toasted bread
Whole grains	Corn, sorghum, millet, oats
Tubers	Sweet potato, potato, taro
Vegetables	Wild vegetables, green vegetable, spinach, green peppers, tomato Chinese cabbage, radish, cucumer, eggplant
Fruit	Apple,pears, peach, apricots, cherries, grapes, bananas, cantaloupe, watermelon, oranges, grapefruit, kiwi,strawberries and et al.
Pickled vegetables	Salted vegetables, Chinese sauerkraut
Mushrooms	Mushroom, shiitakes, enoki
Red meat	Pork, mutton, beef
Poultry and organs	Chicken, duck, liver, animal blood
Processed and cooked meat	Ham and sausage, sauced pork, roast duck
Fish and shrimp	Fish, shrimp
Eggs	Duck eggs, chicken eggs
Seafood	Sea fish, shrimp, crab,squid, jellyfis, shellfish
Bacon and salted fish	Salted meat and duck, salted fish
Salted and preserved eggs	Salted duck and chicken eggs, preserved eggs
milk	Liquid milk, milk powder, yoghurt
Cheese	Cheese
Soya bean and its products	Tofu, dried bean curd, soy milk
Miscellaneous bean	Mung beans, red beans, hemp beans
Fats	Lard, butter
Vegetable oil	Soybean oil, tea oil, rapeseed oil, olive oil
Fast foods	KFC, Mcdonald,fried dough sticks and twists, fried cakes,pizza
Nuts	Walnut, peanuts, almonds, melon seeds
Snacks	Cookies, sachima, bread, cake, ice cream, candy, sweets,potato chips, shrimp roll, popcorn
Chocolates	Chocolates
Honey	Honey, hydromel
Drinks	Coca-cola, sprite, fruit and vegetable drink, fruits juice
Alcoholic beverages	Beer, fruit wine, grape wine
Tea	Tea,scented tea, wong Lo Kat
Coffee	Coffee

Three major dietary patterns were extracted: the traditional southern Chinese dietary pattern (high in refined grains, vegetables, fruits, pickled vegetables, fish and shrimp, bacon and salted fish, salted and preserved eggs, milk, soya bean and its products, miscellaneous bean, fats, and drinks), the Western dietary pattern (high in red meats, poultry and organs, processed and cooked meat, eggs, seafood, cheese, fast foods, snacks, chocolates, alcoholic beverages, and coffee), and the grains-vegetables dietary pattern (high in whole grains, tubers, vegetables, mushrooms, vegetable oil, nuts, honey, and tea).

### Assessment of blood pressure

For blood pressure measurements, participants were first asked to rest in the sitting position for five to ten minutes. Then, three consecutive readings one minutes apart were measured by a trained nurse, with a standard mercury sphygmomanometer (IT168, Yuyue, China), and thereafter the mean of three measurements was considered as the participant’s blood pressure.

### Assessment of biomarker

Blood samples were obtained after 12 h of fasting overnight. After blood was collected, samples were allowed to clot at room temperature for 1–3 h. After clotting, serum was separated by centrifugation for 15 min at 3000r.p.m. Then samples were analyzed in the Medical Center for Physical Examination, Zhejiang Hospital for fasting plasma glucose(FPG), total cholesterol (TC), triglycerides (TG), high-density lipoprotein-cholesterol (HDL-C), low-density lipoprotein-cholesterol (LDL-C), serum uric acid (SUA), alanine aminotransferase (ALT) and asparagine aminotransferase (AST) using the Hitachi 7180 auto-analyzer (Hitachi, Tokyo, Japan).

### Assessment of anthropometric measurements

Height was measured to the nearest 0.1 cm with participants standing without shoes. Weight in light clothes was measured to the nearest 0.1 kg using a digital scale (DHM-200D, China). Body mass index (BMI) was calculated using weight (in kilograms) divided by squared height(in meters). Waist circumference(WC) was measured at the natural waist(minimal circumference between umbilicus and xiphoid process) [18]. Hip circumference was measured to the nearest 0.5 cm at the maximum level over light clothing by using an inelastic plastic tape [19]. All measurements were performed by trained nurses to use standardized procedures.

### Assessment of other variables

Information on physical activity was collected using the International Physical Activity Questionnaire (IPAQ), and expressed as metabolic equivalent hours per week (MET-h/week). Additional information such as smoking habits (never, current, and former smokers), and educational level (primary school or below, middle and high school, junior college or above) were obtained with questionnaires. Total energy intake was estimated through the validated semi-quantitative FFQ, expressed in kilocalorie per day (kcal/day) and categorized according to quartile.

### Definition of T2DM

T2DM was defined as the presence of any one of the following: (1) FPG ≥ 7.0 mmol/L on at least two separate occasions, or an oral glucose tolerance test (OGTT) with a value ≥ 11.1 mmol/L [13]; (2) current use of insulin or oral hypoglycemic agents; or (3) a positive response to the question: Have you ever been diagnosed with diabetes by a doctor?

### Data analysis

The study participants were categorized according to quartile categories of the dietary pattern scores. Results are presented as mean ± SD for continuous variables or number(proportion) of patients for categorical variables. Significant differences in general characteristics across quartile categories of the dietary pattern scores were assessed by using one-way analysis of variance (ANOVA) with Tukey’s post hoc comparisons. The chi-square tests were used to evaluate the significant differences in categorical variables. Logistic regression analysis model was used to evaluate the association of dietary patterns with the risk of T2DM, adjusting for potential confounders. Model 1 was adjusted for sex (male/female) and age(continuous); Model 2 was further education level(<high school, high school, > high school), physical activity level(light, moderate, and heavy), smoking status(never, current, former), BMI. Model 3 was additionally adjusted for total energy intake.All statistical analyses were performed by using version 21.0 of the SPSS software package(SPSS Inc., Chicago, IL, USA), and a 2-tailed *P* < 0.05 was considered statistically significant.

## Results

Overall prevalence of T2DM in our study population was 23.6%.General and clinical characteristics of participants with and without T2DM are shown in Table 2 (*n* = 1918).There were significant differences between participants with and without T2DM by age, educational level, economic income, and the prevalence of obese and hypertension(*P* < 0.05).Participants with T2DM were significantly older (54.82 ± 9.63 vs 51.48 ± 9.56) and higher prevalence of obesity (13.5% vs 5.4%) and hypertension (26.5% vs 21.9%) than those without T2DM.

**Table 2:** General and clinical characteristics of participants with and without T2DM

Variables	Participants with T2DM (*n* = 453)	Participants without T2DM (*n* = 1465)	Significance^*^
Demographic			
Age (years)	54.82 ± 9.63	51.48 ± 9.56	*P* = 0.000
Gender			
Male	253(55.8)	743(50.7)	χ^2^ = 3.652 *P* = 0.056
Female	200(44.2)	722(49.3)	
Smoking status (%)			
Never	342(75.5)	1154(78.8)	χ^2^ = 3.481 *P* = 0.175
Former	13(2.9)	50(3.4)	
Current	98(21.6)	261(17.8)	
Education (%)			
< High school	44(9.8)	264(18.0)	χ^2^ = 23.727 *P* = 0.000
High school	369(81.4)	1125(76.8)	
> High school	40(8.8)	76(5.2)	
Monthly income per person (%)			
≤ 2500 (RMB)	202(44.6)	541(36.9)	χ^2^ = 17.475 *P* = 0.000
2500–4000 (RMB)	218(48.2)	721(49.2)	
> 4000 (RMB)	33(7.2)	203(13.9)	
Obese (%)			
Yes	61(13.5)	79(5.4)	χ^2^ = 33.329
No	392(86.5)	1386(94.6)	*P* = 0.000
Hypertension (%)			
Yes	120(26.5)	321(21.9)	χ^2^ = 4.097
No	333(73.5)	1144(78.1)	*P* = 0.043

Categorical variables are presented as sum and percentages, and continuous variables are presented as Mean ± SD

*Abbreviations*: *MetS* Metabolic syndrome, *T2DM* Type 2 diabetes mellitus

^*^ *P* values for continuous variables (Analysis of variance) and for Categorical variables (chi-square test)

Factor analysis revealed three major dietary patterns(Appendix 2): the traditional southern Chinese dietary pattern (high in refined grains, vegetables, fruits, pickled vegetables, fish and shrimp, bacon and salted fish, salted and preserved eggs, milk, soya bean and its products, miscellaneous bean, fats, drinks), the Western dietary pattern (high in red meats, poultry and organs, processed and cooked meat, eggs, seafood, cheese, fast foods, snacks, chocolates, alcoholic beverages, coffee), and the grains-vegetables dietary pattern (high in whole grains, tubers, vegetables, mushrooms,vegetable oil, nuts, honey, tea), which explained 10.3, 8.5 and 6.8% of the dietary intake variance, respectively. The factor-loading matrixes for these dietary patterns are shown in Table 3.

**Table 3:** Factor-loading matrix for the three dietary patterns^a^

Food groups	Dietary patterns		
	Traditional southern Chinese	Western	Grains-vegetables
Refined grains	0.411	–	–
Whole grains	–	–	0.534
Tubers	–	–	0.471
Vegetables	0.407	–	0.638
Fruit	0.462	–	–
Pickled vegetables	0.509	–	–
Mushrooms	–	–	0.664
Red meat	–	0.563	–
Poultry and organs	–	0.502	–
Processed and cooked meat	–	0.520	–
Fish and shrimp	0.486	–	–
Eggs	–	0.346	–
Seafood	–	0.417	–
Bacon and salted fish	0.529	–	–
Salted and preserved eggs	0.414	–	–
Milk	0.360	–	
Cheese	–	0.315	
Soya bean and its products	0.400	–	–
Miscellaneous bean	0.414	–	–
Fats	0.447	–	
Vegetable oil	–	–	0.392
Fast foods	–	0.407	–
Nuts	–	–	0.303
Snacks	–	0.517	–
Chocolates	–	0.435	–
Honey	–	–	0.595
Drinks	0.472	–	
Alcoholic beverages	–	0.301	–
Tea	–	–	0.357
Coffee	–	0.390	–
Variance of intake explained (%)	10.3	8.5	6.8

^a^Absolute values < 0.4 were excluded for simplicity

General characteristics of study participants across quartiles of the major dietary pattern scores are shown in Table 4. Subjects in the fourth quartile of the traditional southern Chinese dietary pattern were older, female, less likely to be smokers and have a higher education level.In addition, compared with those in the lowest quartile, individuals in the highest quartile of the Western dietary pattern were more likely to be males, smoker, younger, and higher BMI, WC, and economic income, and had a higher prevalence of obesity, hypertension and T2DM. Subjects in the highest quartile of the grains-vegetables dietary pattern were older, female, less likely to be smokers, and lower BMI, WC, WHR and had significantly lower prevalence of obesity, hypertension and T2DM and higher economic income.

**Table 4:** General characteristics of study participants across quartiles of the major dietary pattern scores

	Traditional southern Chinese dietary pattern score		P*	Western dietary pattern score		P*	Grains-vegetables dietary pattern score		P*
	Q1 (lowest) (*n* = 479)	Q4 (highest) (*n* = 480)		Q1 (lowest) (*n* = 479)	Q4 (highest) (*n* = 479)		Q1 (lowest) (*n* = 480)	Q4 (highest) (*n* = 479)	
Age (y)	50.3 ± 0.2	51.8 ± 0.3	**< 0.001**	51.5 ± 0.2	49.7 ± 0.2	**< 0.001**	50.0 ± 0.2	51.9 ± 0.3	**< 0.001**
BMI(kg/m^2^)	24.39 ± 3.11	24.93 ± 2.96	0.225	24.27 ± 2.81	25.10 ± 3.12	**< 0.05**	25.33 ± 2.85	24.11 ± 2.86	**< 0.01**
WC(cm)	84.79 ± 9.03	85.78 ± 8.65	0.577	84.05 ± 8.61	87.05 ± 8.24	**< 0.01**	87.68 ± 8.92	82.65 ± 8.35	**< 0.01**
WHR	0.87 ± 0.06	0.88 ± 0.06	0.683	0.87 ± 0.08	0.89 ± 0.06	0.068	0.89 ± 0.06	0.86 ± 0.08	**< 0.05**
Obesity (%)	78(16.3)	70(14.6)	0.483	56(11.7)	88(18.4)	**< 0.05**	80(16.7)	43(9.0)	**< 0.01**
Hypertension (%)	152(31.7)	167(34.9)	0.387	114(23.8)	156(32.6)	**< 0.05**	175(36.5)	121(25.2)	**< 0.01**
T2DM (%)	98(20.4)	103(21.5)	0.689	79(16.5)	119(24.8)	**< 0.01**	94(19.6)	64(13.3)	**< 0.05**
Gender (%)			**< 0.001**			**< 0.05**			**< 0.001**
Male	325(67.8)	240(50.1)		256(53.4)	301(62.8)		296(61.8)	175(36.5)	
Female	155(32.2)	239(49.9)		223(46.6)	178(37.2)		183(38.2)	305(63.5)	
Smoking status (%)			**< 0.05**			**< 0.001**			**< 0.05**
Never	405(84.3)	358(74.7)		358(74.7)	319(66.6)		372(77.7)	415(86.4)	
Current	69(14.4)	113(23.6)		76(15.9)	140(29.2)		78(16.3)	40(8.3)	
Former	6(1.3)	8(1.7)		45(9.4)	20(4.2)		29(6.0)	25(5.3)	
Education (%)			**< 0.001**			**< 0.001**			0.570
< High school	164(34.2)	78(16.3)		103(21.5)	95(19.8)		127(26.6)	112(23.3)	
High school	147(30.6)	137(28.6)		177(37.0)	201(42.0)		148(30.9)	147(30.6)	
> High school	169(35.2)	264(55.1)		199(41.5)	183(38.2)		204(42.5)	221(46.1)	
The average monthly income per person (%)			0.889			**< 0.05**			**< 0.05**
< 2000(RMB)	124(25.8)	130(27.1)		137(28.6)	97(20.3)		161(33.6)	121(25.2)	
2000–4000(RMB)	201(41.9)	202(42.2)		193(40.3)	188(39.2)		192(40.1)	183(38.1)	
> 4000(RMB)	155(32.3)	147(30.7)		149(31.1)	194(40.5)		126(26.3)	176(36.7)	
Physical activity (%)			0.109			**< 0.05**			0.361
Light	387(80.6)	417(87.1)		376(78.5)	404(84.3)		379(79.1)	378(78.7)	
Moderate	73(15.2)	49(10.2)		78(16.3)	65(13.6)		89(18.6)	81(16.9)	
Vigorous	20(4.2)	13(2.7)		25(5.2)	10(2.1)		11(2.3)	21(4.4)	
Total energy(kcal/d)	2663.7 ± 195.6	2446.5 ± 265.5	0.379	2230.1 ± 280.7	2734.9 ± 268.4	**< 0.01**	2519.3 ± 230.0	2239.4 ± 195.1	**< 0.05**

Categorical variables are presented as sum and percentages, and continuous variables are presented as Mean ± SD

*Abbreviations*: *BMI* Body mass index, *WC* Waist circumference, *WHR* Waist hip rate, *T2DM* Type 2 diabetes mellitus

*P values for continuous variables (Analysis of variance) and for Categorical variables (chi-square test)

The relationship between major dietary patterns and T2DM risk by logistic regression analysis is shown in Table 5. After adjustment for sex, age, education level(<high school, high school, >high school), physical activity level(light, moderate, and heavy), smoking status(never, current, former), BMI and total energy intake, subjects in the highest quartile of the grains-vegetables dietary pattern score had lower odds of the T2DM(OR = 0.72; 95% CI:0.596–0.952; *P* = 0.03) than did those in the lowest quartile, whereas those in the highest quartile of the western dietary pattern score had greater odds of T2DM(OR = 1.28; 95% CI:1.103–1.697; *P* = 0.029) than did those in the lowest quartile. Moreover, the traditional southern Chinese pattern showed no association with the risk of T2DM.

**Table 5:** Multivariable adjusted ORs and 95% CIs for T2DM across the quartile (Q) categories of dietary pattern scores in Zhejiang Province, China

	Traditional southern Chinese pattern score			Western pattern score			Grains-vegetables pattern score		
	Q1 (*n* = 479)	Q4 (*n* = 480)	*p*	Q1 (*n* = 479)	Q4 (*n* = 479)	*p*	Q1 (*n* = 480)	Q4 (*n* = 479)	*p*
Model 1	1.00	1.27 (1.026,1.537)	0.04	1.00	2.17 (1.462, 4.063)	0.000	1.00	0.50 (0.364, 0.682)	0.000
Model 2	1.00	1.13 (0.953, 1.417)	0.247	1.00	1.63 (1.317, 2.059)	0.003	1.00	0.67 (0.524, 0.857)	0.000
Model 3	1.00	1.09 (0.825, 1.495)	0.440	1.00	1.28 (1.103, 1.697)	0.02	1.00	0.72 (0.596, 0.952)	0.04

Model 1:adjusted for sex and age; Model 2: further adjusted for education level (<high school, high school, >high school), physical activity level (light, moderate, and heavy), smoking status (never, current, former) and BMI; Model 3: additionally adjusted for total energy intake. Q4: the highest quartile of dietary patterns, Q1: the lowest quartile of dietary patterns (reference)

*OR* Odds ratio, *95%CI* 95% confidence interval, *T2DM* Type 2 diabetes mellitus, *CI* confidence interval

## Discussion

Limited attention has been given to the role of dietary patterns in the development of T2DM in a Chinese population.To the best of our knowledge, this study is the first to clarify the associations between major dietary patterns and the risk of T2DM in a middle-aged Chinese population. In the present study, our results indicated that the grains-vegetables dietary pattern was associated with a significantly decreased risk, while the Western dietary pattern was associated with increased risk for T2DM. Moreover, no significant association was found between the traditional southern Chinese dietary pattern and risk of T2DM.

In our analyses, no significant association was found for the traditional southern Chinese dietary pattern and the risk of T2DM. This finding is not line with a previous study [20] that has reported the favorable effect of dietary patterns high in fruits and vegetables on the prevention of T2DM. There are several possible explanations for this result. First, high intakes of refined grains have been found to increase the risk of obesity and hypertension, which are the important risk factors for T2DM [21]. Second, pickled vegetables in the traditional southern Chinese pattern contain huge amounts of salt. Our previous study also found that the high-salt dietary pattern could increase the risk of hypertension [22]. It is known that hypertension has been acknowledged as an important risk factor for T2DM. In contrast, previous studies [23, 24] have indicated that antioxidants (e.g. vitamin C, E) and dietary fiber abundant in fruits are associated with decreased risk of obesity and hypertension, important risk factors for T2DM. Furthermore, the lack of association between this pattern and T2DM risk could also be due to reverse causal association. Participants with risk of T2DM may be advised to change dietary habits, limiting the intake of foods containing high amounts of sugar, during routine examinations. In summary, these possibilities could not be excluded in this study.

A Western dietary pattern(high in refined grains, red meat, processed meat) was significantly associated with increased risk of T2DM. Our finding was consistent with the previous observations [25]. The positive association between the western dietary pattern and the risk of T2DM may partly be attributed to unhealthy dietary constituents, such as refined grains,red meat and fats/oils in this pattern. Firstly, high consumption of meat, especially red meat containing high amounts of saturated fat and cholesterol, was associated with an increased risk of obesity, an important risk factor for T2DM [15]. Meanwhile, higher intakes of red meat may result in more absorption of haem iron [25]. Previous studies have demonstrated that body iron overload may promote insulin resistance and increase the risk of T2DM [26]. Secondly, processed meat often contains high concentrations of nitrates or nitrites, and nitrosamine compounds, which are thought to increaseT2DM risk [25]. Thirdly, advanced glycation of high-fat products and meats can enhance oxidative stress and inflammatory factors that may be accompanied with insulin resistance and increase the possibility of developing T2DM [27]. Fourthly, in the European Prospective Investigation into Cancer and Nutrition-Potsdam Study, higher intakes of meat are related to risk of weight gain, a known risk factor for T2DM [28].

The grains-vegetables dietary pattern was characterized by high consumption of whole grains, vegetables and beans.In the present study, we observed the significantly inverse association between grains-vegetables dietary pattern and the risk of T2DM. Our results were in agreement with previous a systematic review and meta-analysis, which demonstrated an inverse relationship between whole grain intake and the risk of T2DM [29]. There are several possible explanations for the inverse association. Firstly, dietary fiber intake has been found to be inversely associated with insulin resistance, which has been reported to be the risk factor for T2DM [30]. Secondly, some foods in the grains-vegetables dietary pattern have a low glycemic load, which have been documented to be associated with a lower risk of T2DM [31].Thirdly, our finding also indicated that the study participants who belonged to the fourth quartile of grains-vegetables dietary pattern had a higher level of physical activity, compare to those in the lowest quartile. A previous study suggested that physical activity as a form of energy expenditure was closely related to a decreased risk of obesity [32], an important risk factor for T2DM. Furthermore, green tea has also been suggested to decrease the risk of type 2 diabetes mellitus and is associated with decreased insulin resistance [33].

### Strengths and limitations

The present study holds several strengths. Firstly, to our knowledge, this is the first study reporting the relations between dietary patterns and T2DM risk among the middle-aged adults in China. Our findings provided valuable information for the primary prevention of T2DM through the dietary modifications in a middle-aged Chinese population. Secondly, diets were measured with the use of a semi- quantitative FFQ that were validated against multiple-day diet records; This tool enabled us to capture more reliable information on usual dietary intake of individuals during the past year. Thirdly, in our analyses, we also have adjusted for some known and proposed potential confounders in the statistical model. However, some potential limitations warrant mention. Firstly, given the cross-sectional design, we are unable to evaluate the causal associations between dietary patterns and the risk of T2DM.Thus, our findings need to be confirmed in future prospective study. Secondly, factor analysis (principal component) which was used for data reduction has limitations. For example, grouping of different food items and the definition of the dietary patterns, including determination of number of factors, the type of rotation, as well as the interpretation and naming of the factors, involving subjective decisions [34]. Thirdly, the study sample was not necessarily representative of all the general populations, which may limit the generalizability of our findings.

## Conclusions

In conclusion, we found that the Western dietary pattern was associated with increased risk, whereas grains-vegetables dietary pattern was associated with reduced risk of T2DM among Chinese adults aged 45–59y. Although future prospective study are needed to ascertain the associations between dietary patterns and T2DM risk, our findings have added to the growing body of literature that shows that higher consumption of whole grains, tubers, and vegetables are likely beneficial for the prevention of T2DM, while the higher consumption of red meats, poultry and organs, processed and cooked meat may increase the risk of T2DM.

## Figures and Tables

**Fig. 1:**
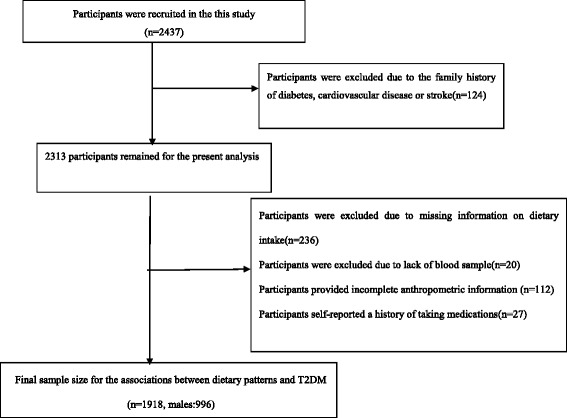
Flow chart of article screening and selection process

## Appendix 2

**Table 6:** Foods included in the three dietary patterns identified

Dietary patterns	Food groups
traditional southern Chinese	Refined grains, vegetables, fruits, pickled vegetables, fish and shrimp, bacon and salted fish, salted and preserved eggs, milk, soya bean and its products, miscellaneous bean, fats, drinks
Western	Red meats, poultry and organs, processed and cooked meat, eggs, seafood, cheese, fast foods, snacks, chocolates, alcoholic beverages, coffee
Grains-vegetables	whole grains, tubers, vegetables, mushrooms,vegetable oil, nuts, honey, tea

## Abbreviations

ALT: Alanine aminotransferase
AST: Asparagine aminotransferase
BMI: Body mass index
CI: Confidence interval
FFQ: Food frequency questionnaire
FPG: Fasting plasma glucose
HDL-C: High-density lipoprotein-cholesterol
IPAQ: International physical activity questionnaire
LDL-C: Low-density lipoprotein-cholesterol
OGTT: Oral glucose tolerance test
OR: Odds ratio
SUA: Serum uric acid
T2DM: Type 2 diabetes mellitus
TC: Total cholesterol
TG: Triglycerides
WC: Waist circumference
